# Using a behaviour change techniques taxonomy to identify active ingredients within trials of implementation interventions for diabetes care

**DOI:** 10.1186/s13012-015-0248-7

**Published:** 2015-04-23

**Authors:** Justin Presseau, Noah M Ivers, James J Newham, Keegan Knittle, Kristin J Danko, Jeremy M Grimshaw

**Affiliations:** Institute of Health & Society, Faculty of Medical Sciences, Newcastle University, Newcastle upon Tyne, UK; Family Practice Health Centre, Women’s College Research Institute, Women’s College Hospital, Toronto, Canada; Women’s College Research Institute, Women’s College Hospital, Toronto, Canada; Institute for Health Systems Solutions and Virtual Care, Women’s College Hospital, Toronto, Canada; Department of Family and Community Medicine, University of Toronto, Toronto, Canada; Institute of Health Policy Management and Evaluation, Dalla Lana School of Public Health, University of Toronto, Toronto, Canada; Department of Social Research, University of Helsinki, Helsinki, Finland; Clinical Epidemiology Program, Ottawa Hospital Research Institute, Ottawa, Canada; School of Epidemiology, Public Health and Preventive Medicine, Faculty of Medicine, University of Ottawa, Ottawa, Canada

**Keywords:** Behaviour change, Taxonomy, Diabetes, Quality improvement, Techniques, Intervention content

## Abstract

**Background:**

Methodological guidelines for intervention reporting emphasise describing intervention content in detail. Despite this, systematic reviews of quality improvement (QI) implementation interventions continue to be limited by a lack of clarity and detail regarding the intervention content being evaluated. We aimed to apply the recently developed Behaviour Change Techniques Taxonomy version 1 (BCTTv1) to trials of implementation interventions for managing diabetes to assess the capacity and utility of this taxonomy for characterising active ingredients.

**Methods:**

Three psychologists independently coded a random sample of 23 trials of healthcare system, provider- and/or patient-focused implementation interventions from a systematic review that included 142 such studies. Intervention content was coded using the BCTTv1, which describes 93 behaviour change techniques (BCTs) grouped within 16 categories. We supplemented the generic coding instructions within the BCTTv1 with decision rules and examples from this literature.

**Results:**

Less than a quarter of possible BCTs within the BCTTv1 were identified. For implementation interventions targeting providers, the most commonly identified BCTs included the following: adding objects to the environment, prompts/cues, instruction on how to perform the behaviour, credible source, goal setting (outcome), feedback on outcome of behaviour, and social support (practical). For implementation interventions also targeting patients, the most commonly identified BCTs included the following: prompts/cues, instruction on how to perform the behaviour, information about health consequences, restructuring the social environment, adding objects to the environment, social support (practical), and goal setting (behaviour). The BCTTv1 mapped well onto implementation interventions directly targeting clinicians and patients and could also be used to examine the impact of system-level interventions on clinician and patient behaviour.

**Conclusions:**

The BCTTv1 can be used to characterise the active ingredients in trials of implementation interventions and provides specificity of content beyond what is given by broader intervention labels. Identification of BCTs may provide a more helpful means of accumulating knowledge on the content used in trials of implementation interventions, which may help to better inform replication efforts. In addition, prospective use of a behaviour change techniques taxonomy for developing and reporting intervention content would further aid in building a cumulative science of effective implementation interventions.

**Electronic supplementary material:**

The online version of this article (doi:10.1186/s13012-015-0248-7) contains supplementary material, which is available to authorized users.

## Background

Evidence from trials testing the effectiveness of interventions to improve quality of care can help researchers, practitioners, and decision-makers better promote the integration of research findings into practice. Implementation interventions can be complex [1], involving a single or multiple components delivered through a range of modalities across various settings. Characterising the content used in an implementation intervention is fundamental for reporting, replicating, and synthesising evidence [2-5]. However, without a clear and shared understanding of how to best describe the content of implementation interventions, a number of risks emerge: a) using the same content description to represent different types of content, b) using different terms to represent the same content [6,7], c) using levels of description that are not sufficiently specific to allow replication [8], d) repetition/reinvention without progress [9], and e) missed opportunities to draw on techniques used effectively in other settings. Describing implementation interventions using a comprehensive classification system with agreed definitions could address these issues.

Intervention content classification systems are not new. The Effective Practice and Organisation of Care (EPOC) group maintains a list of implementation strategies, which has been used to classify intervention content in systematic reviews of interventions designed to improve the delivery, practice, and organisation of healthcare services. A recently published systematic review in the Lancet of 142 randomised trials of implementation interventions for diabetes used a subset of the EPOC taxonomy based on an earlier version of the review published in JAMA [10]. The Lancet review classified implementation intervention content into 12 strategies [audit and feedback, case management, clinician education, clinician reminders, continuous quality improvement (QI), electronic patient registry, facilitated relay, financial incentives, patient education, patient reminders, self-management, and team changes] organised into three broader categories (health system, healthcare provider, and patient). The review showed that implementation interventions can be effective in changing both processes and clinical outcomes in diabetes care, with various EPOC strategies showing similar effects and unexplained heterogeneity. While an important step forward in providing a common language, the EPOC taxonomy has some limitations. Specifically, a) it confounds content, mode of delivery, and provider [2,5], e.g. an educational meeting is a mode of delivery which can involve a range of different behaviour change techniques delivered in different ways; b) categories provide variable levels of detail; and c) strategies in the EPOC taxonomy predominantly target changing resources and opportunities as means of implementing desired change.

Herein, we use ‘implementation intervention’ as an overarching term to enable a distinction from its constituent components, which may be described as particular strategies and/or change techniques. For example, the EPOC taxonomy may be considered as a list of implementation strategies. It has been suggested that description at the strategy level can act as a helpful shorthand if used reliably but may be insufficient for replication if the strategy is not described in sufficient detail [3,8]. To better characterise the detail of implementation interventions, there is a need for greater clarity of its specific, active ingredients. The appropriate classification system depends, of course, on the goals of communication. Consider the example of cardiac rehabilitation. There is ample evidence that cardiac rehabilitation improves patient outcomes [11], but suboptimal referral [12,13] and participation [14] both limit the extent of impact. For the referring physician, it might be enough to know that a referral to cardiac rehabilitation has been made and the patient is actively participating. However, to achieve best outcomes, cardiac rehabilitation programme administrators must ensure each component of cardiac rehabilitation (e.g. lifestyle counselling, supervised exercise) is delivered as effectively as possible. Therefore, programme administrators must identify and implement specific, active ingredient to optimise patient outcomes [15,16].

Behaviour change approaches to implementation science provide an opportunity to draw upon decades of applied research in behavioural medicine and social and health psychology regarding the techniques that can be used to change behaviour. A behaviour change technique (BCT) is defined as “an observable, replicable, and irreducible component of an intervention designed to alter or redirect causal processes that regulate behaviour; that is, a technique is proposed to be an ‘active ingredient’ (p. 23)” [8]. Describing an implementation intervention in terms of BCTs, i.e. active ingredients, may provide a more useful level of detail for synthesis, comparison, and replication of trials of implementation interventions.

The last decade has seen the development of comprehensive taxonomies of BCTs that can be used to classify active ingredients of interventions using agreed definitions. Initially, taxonomies were limited to applications in particular behavioural areas focusing on patients and the public, including physical activity and healthy eating [17,18], alcohol consumption [19], and smoking cessation [20]. Such taxonomies have improved our understanding of the content of interventions in those areas, providing opportunities to synthesise evidence at a BCT level. For example, Dombrowski and colleagues [21] used a BCT taxonomy to classify content of interventions for obese adults with additional risk factors and showed that specific BCTs were associated with intervention effectiveness, including ‘self-monitoring’, ‘relapse prevention’, and ‘prompting practice’. Such analyses have been conducted in a range of health behaviour contexts [22-24]. More recently, a comprehensive BCT taxonomy has been developed through an international consensus process. The resulting taxonomy, the Behaviour Change Techniques Taxonomy version 1 (BCTTv1), includes 93 BCTs grouped within 16 categories with detailed definitions of each [8].

Existing applications of BCT taxonomies to the implementation science literature have been limited to subsets of BCTs to understand commonly tested implementation interventions, e.g. within the audit and feedback literature [25-27]. Another use of BCT taxonomies has been to provide a classification system for assessing intervention fidelity of intervention delivery [28]. However, to our knowledge, no previous studies have examined whether the BCT approach can be applied to the implementation literatures, which tend to target multiple different recipients. For example, in the diabetes QI literature, multi-faceted initiatives often attempt to simultaneously act upon patients, health professionals, and/or the system in which they interact with each other. We aimed to explore whether the BCTTv1 can be used to identify the active ingredients of existing implementation interventions described at patient, provider, and system levels.

## Method

### Design and study selection

We conducted a secondary analysis of a subset of trials reported in a review of trials of QI interventions for diabetes [10]. A total of 23 trials were selected from 142 (16%) for coding. To ensure representative diversity of content, a researcher (KJD) randomly selected two studies using an online list randomiser (www.random.org) from each of the 12 EPOC taxonomy categories that had previously been used to categorise intervention content (only one trial was available for financial incentives). As interventions typically included multiple EPOC taxonomy strategies (e.g. clinician education and team changes), some strategies were represented more than twice. BCT coders were blind to the original EPOC taxonomy coding of the papers selected.

### Types of studies

Studies were randomised or cluster-randomised controlled trials of interventions for improving the management of diabetes included in Tricco et al. [10].

### Behaviour change technique coding

Three psychologists (JP, JJN, KK) independently coded all 23 trials using the BCTTv1 definitions and examples as the initial coding manual using nVivo version 9 (QSR International Pty Ltd, Doncaster, Australia, 2010). The coders were experienced in using BCT taxonomies and in intervention development in health psychology and implementation science contexts. Content was coded within reports of intervention and control group content descriptions from source papers. BCTs were coded for two recipients of the intervention (action targets), a) BCTs targeting existing healthcare providers’ behaviour and b) BCTs targeting patients’ behaviour, where reported. System-level interventions were coded as targeting healthcare providers’ and/or patients’ behaviour. All 93 BCTs were considered for each of the 23 trials. Each coder added clarification to the coding manual to reflect any assumptions made for further discussion.

Following coding, nVivo was used to generate a study-by-study report of all BCT coding, which flagged discrepancies to be discussed by the three coders. Each discrepant BCT code was resolved and noted, with corresponding changes made to the coding manual to address the sources of discrepancies. When only one or two of the three coders coded a BCT, it was flagged for discussion as a potential discrepancy, which occurred across all 23 trials. Discussion consisted of the coder(s) who had identified the BCT justifying their reasoning to those who had not identified the BCT. When coders could not reach consensus, the example was discussed with the wider remaining team (NMI, KJD, JMG) who reviewed and further discussed the coded BCT data. Consensus was preferred over formal agreement statistics given the proof of concept nature of the study, and the need for developing a coding manual with sufficient breadth to account for the range of interventions tested.

### Coding assumptions

Four assumptions were made when coding. First, all interventions (including those involving the system level) were coded with the assumption that BCTs operated through targeting the behaviour of providers or their patients. Second, if specific underlying target behaviours were not clear, BCTs were coded at the more general behavioural level of ‘improving processes of care’. Third, when interventions were described as involving the provision of ‘education’ without any further detail, we assumed that any educational session within an implementation intervention would involve the following two BCTs at minimum: *information about health consequences* and *instruction on how to perform the behaviour*; although additional BCTs would be coded if judged to be present. This assumption was made to ensure acknowledgement of a minimum content of the educational strategy. Fourth, when interventions were described as providing ‘training’ without further detail, we assumed that unless otherwise stated, any implementation intervention describing a training session would at least provide *instruction on how to perform the behaviour*. The latter two assumptions are a departure from more conservative approaches typically applied in the use of the BCTTv1. However, given the prevalence of education and training in this literature, we decided, in conjunction with the expertise on the team, that it would be a misrepresentation to omit such content.

### Coding manual development

The BCTTv1 provides detailed definitions of each of the 93 BCTs and includes examples of each. However, most examples apply to health behaviours rather than healthcare provider behaviours. To aid in the development of future iterations of the BCTTv1, we developed coding rules and examples to supplement those already provided by the BCTTv1 which are specific to provider behaviours as these are under-represented as examples in the BCTTv1 (see Additional file 1). We added coding rules to 26 BCTs covering what to code and what not to code, based on discussions between coders during discrepancy-resolution meetings.

### Data analysis

We quantified the frequency of identified BCTs and each category of BCT across studies targeting change in provider and patient behaviour.

## Results

### Types of behaviours targeted

The vast majority of implementation interventions focused on changing multiple behaviours in clinicians and patients, often described at a higher-level description than at specific behaviours. For example, most implementation interventions targeted a range of processes of care at once, often focusing on evidence-based care processes and outcomes.

### Implementation interventions targeting provider behaviours

**Figure 1:**
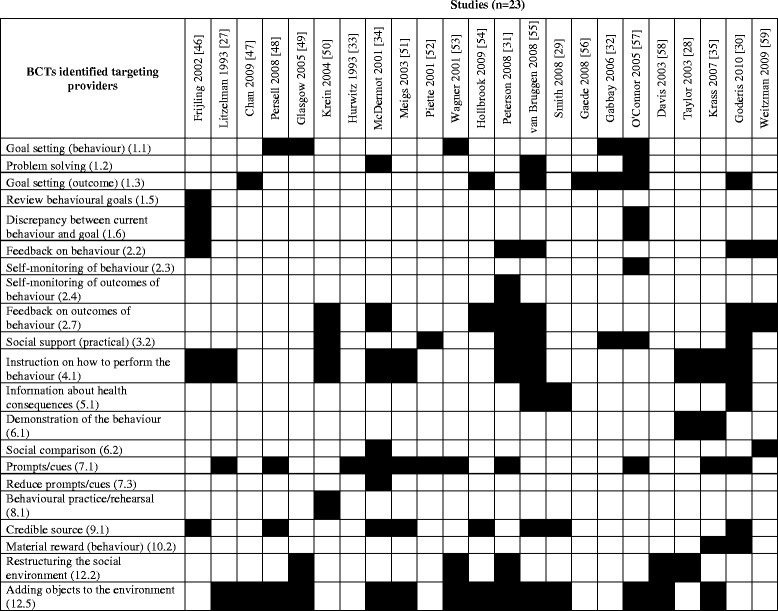
List of BCTs coded targeting provider behaviours in intervention groups.

**Figure 2:**
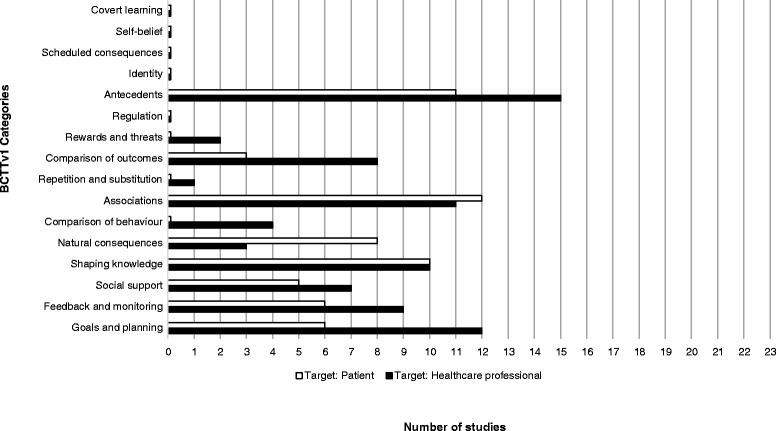
Frequency of coding of each BCTTv1 category out of 23 studies for healthcare professionals and patients.

Figure 1 shows the BCTs coded for each of the 23 trials. Overall, 21 out of a possible 93 BCTs (22.5%) were identified as targeting change in provider behaviours. As seen in Figure 2, at least one BCT was identified in 11 of 16 possible categories of BCTs, though no BCTs were identified within *covert learning*, *self*-*belief*, *scheduled consequences*, *identity*, or *regulation* categories. The most frequently coded BCTs included the following:

*Adding objects to the environment* (14 studies) e.g. “The systems intervention, designed to direct health care providers’ attention to the prevention of patient-specific risk factors, consisted of colorful folders with foot decals to identify intervention patients” [29].

*Prompts/cues* (11 studies) e.g. “[…] pharmacists were contacted regularly by telephone and sent monthly newsletters to keep them informed and motivated” [30].

*Instruction on how to perform the behaviour* (10 studies) e.g. “All intervention pharmacists received a diabetes education manual for self-directed learning [15] and also attended a 2-day workshop. The workshop comprised a mixture of lectures on diabetes, pharmacotherapy, dietary management […]; insulin injection technique and devices; and blood pressure measurement” [31].

*Credible source* (8 studies) e.g. “The resulting messages were brief, fully referenced (including links to longer abstracts that highlighted methodological quality and results and to the full text of publications), and linked to local (e.g. Institute for Clinical Systems Improvement) and national guidelines” [32].

*Goal setting (outcome)* (7 studies) e.g. “Targets were set at 7% for HbA1c, 130 mmHg for SBP and 100 mg/dl” [33].

*Feedback on outcome of behaviour* (7 studies) e.g. “Audit and review monthly. Provide feedback to improve progress” [34].

*Social support* (*practical*) (7 studies) e.g. “[nurse case manager provided] surveillance of patients, including phone calls to patients, referred patients to a certified diabetes nurse educator or a dietitian where appropriate” [35].

### Implementation interventions also targeting patient behaviours

**Figure 3:**
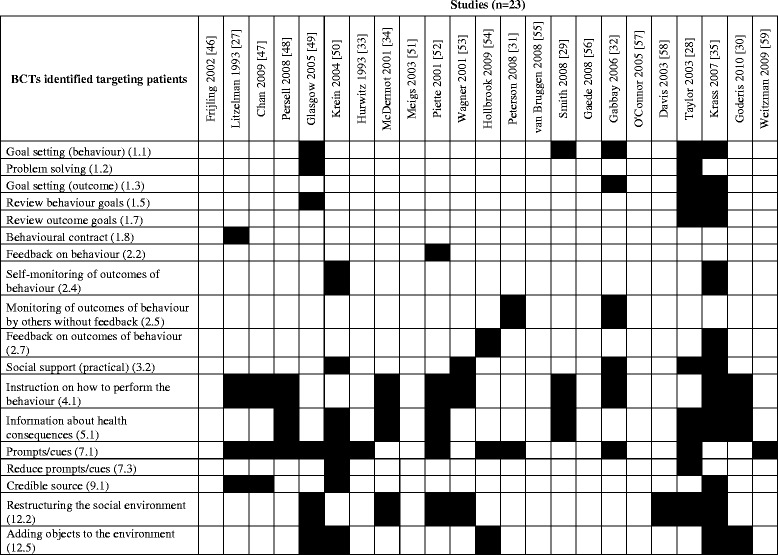
List of BCTs coded targeting patients’ behaviours in intervention groups.

Figure 3 shows the BCTs coded for each of the 23 studies for patients. Out of 93 possible BCTs, we identified 18 (19.4%) targeting changes in patients’ behaviour. As seen in Figure 2, at least one BCT was identified in 8 of 16 possible categories, though no BCTs were identified involving the categories of *covert learning*, *self*-*belief*, *scheduled consequences*, *identity*, *regulation*, *rewards and threats*, *repetition and substitution*, or *comparison of behaviour*. Also seen in Figure 2, and similar to the results of interventions targeting provider behaviour, the most frequently identified BCTs targeting patient behaviours included:

*Prompts/cues* (12 studies) e.g. “The hub of the prompting system is a database which sends requests to patients asking them to provide blood and urine samples for random plasma glucose, glycated haemoglobin, and albumin estimations. […] Patients not already under the care of a hospital eye clinic also receive an annual eye test prompt” [36].

*Instruction on how to perform the behaviour* (10 studies) e.g. “[…] patient education session with one to four patients, covering appropriate foot-care behaviors and footwear” [29].

*Information about health consequences* (8 studies) e.g. “We developed an electronic library of messages using systematic reviews of the best available research on use of aspirin; use of ACE inhibitors and angiotensin receptor blockers (ARBs); management of dyslipidemia, hypertension, chronic heart failure, and nicotine dependence; glycemic control; and diet and exercise. During the clinical encounter, the primary care team and the patient decided how to proceed after reviewing the message and considering its relevance and appropriateness” [32].

*Restructuring the social environment* (7 studies) e.g. “At the same time, a new diabetes outreach service (comprising a diabetologist, nutritionist, podiatrist and diabetes healthcare worker) started which visited all 21 centres equally during the 12-month study period” [37].

*Adding objects to the environment* (5 studies) e.g. “[…] we organized the distribution of printed educational brochures, pedometers and home blood glucose material (HBGM)” [33].

*Goal setting (behaviour)* (5 studies) e.g. *“*Individual goals were set at each visit, documented on a worksheet and reviewed at each subsequent visit” [31].

## Discussion

### Summary

Using the BCTTv1 to code a random sample of randomised trials of diabetes implementation interventions, we showed that it is feasible and useful to apply the BCT taxonomy to characterise the potentially active ingredients within implementation interventions. With less than a quarter of all possible BCTs identified in this sample of trials of implementation interventions, there remains opportunity for the development of novel initiatives that incorporate BCTs with potential for impact but which appear to be underutilised in trials of implementation interventions. Our codebook can provide a foundation for future research in this area, including applications to other implementation contexts.

Taxonomies such as the EPOC taxonomy and the BCTTv1 provide a common language and definitions for understanding the content of implementation interventions across contexts allowing the capacity to draw on insight from a range of populations to inform the design of future interventions. Many of the interventions coded were of a scope, level of detail, and rigour in trial methodology that would serve as examples of good practice. Whereas the EPOC taxonomy provides a summary description of the strategy, the BCTTv1 provides a degree of granularity better suited to describing the active ingredients. As implementation science seeks to build cumulative knowledge regarding how to best design and deliver implementation interventions [38], there is reason to believe that content identification of such interventions in terms of BCTs may offer greater hope for replication and novel insights regarding how to optimise implementation interventions.

### Provider behaviour change

Tentatively, the most common BCTs identified in trials of diabetes implementation interventions to change provider behaviour appear to be *Goal setting (outcome)*, *Feedback on behaviour*, *Credible source*, *Prompts/cues*, *Instruction on how to perform the behaviour*, *Adding objects to the environment*, and *Social support (practical)*. The question is no longer whether BCTs can be used to characterise the detail of trials of implementation interventions; the answer is clearly yes. What is perhaps more relevant is to investigate contextual, theoretical, delivery, and behavioural effect modifiers for a given BCT to help inform the development of future implementation interventions. For example, the extent to which BCTs utilised in this literature address known determinants of provider behaviour requires further study*,* as does the clarification of the moderating role of the actor, the action target, the temporality, and the dose [3] of the BCTs used.

### Patient behaviour change

It is noteworthy that in the sample of 23 studies that we examined, similar BCTs were identified for targeting patients’ behaviour as those targeting clinicians’, though many of the self-regulatory techniques identified to target patient behaviour were identified less frequently when targeting clinician behaviour. Tentatively, the most frequently identified BCTs targeting patients included *Goal setting (behaviour)*, *Prompts/cues*, *Instruction on how to perform the behaviour*, *Information about health consequences*, *Adding objects to the environment*, and *Restructuring the social environment*. However, the 23 studies identified may not be fully representative of all studies targeting patients.

Many of the techniques for changing patient behaviour identified in the present study have also been highlighted in a recent broader review of interventions to change patients’ physical activity behaviour in type 2 diabetes, which identified a number of specific BCTs as being associated with a greater change in HbA1c [24]. Future implementation interventions involving a component of patient behaviour change could be well-served to consider incorporating these BCTs.

### Coding system-level implementation interventions using the BCTTv1

Quality improvement is often a multilevel endeavour [39,40]. While behaviour change approaches are sometimes criticised for focusing on the individual rather than higher levels, this is a false dichotomy. Individuals at multiple levels need to change what they do for implementation to take place. While most implementation interventions report the sharp end of the change at the clinician and patient level, better reporting of who needs to do what, differently [41] at higher levels, would provide a comprehensive perspective that accounts for behaviour change at multiple levels. Such an approach would be consistent with, and contribute to, describing the foundational components of implementation intervention programme theory, further aiding in the generalizability of implementation trials [42]. In practice, reports of system-level strategies do not always include this level of detail. One way to account for this is to assume, as we have, that the behaviour of the clinicians providing care within the system is an ultimate target of many system-level efforts. Given the outcomes in the trials reviewed herein are described in terms of changes to provider and/or patient outcomes, this seemed a reasonable assumption to make. Although it was not possible to directly test this assumption in the present study, future research could test this, assuming the key action targets across levels are made clear. Reporting of key action targets [3] across multiple levels would help to clarify who was targeted at each level and provide the capacity to decompose system-level changes into who did something differently. This may help to more fully describe and replicate strategies that target those other than clinicians and patients.

Other content classification systems such as the EPOC taxonomy include system-level strategies (e.g. continuous QI) that are themselves not particularly well-defined and in some ways overlap with other strategies such as audit and feedback [43]. A BCT approach may provide one way to more explicitly characterise the content of such interventions with greater clarity. Similarly, the BCTTv1 also provides considerable depth regarding different BCTs related to incentives, reward, and threat. The result may provide a more nuanced perspective that draws on behavioural theory to categorise and develop implementation interventions involving financial incentives and rewards that may aid replication.

System-level implementation interventions are codable within the BCTTv1. The most commonly coded BCT for system-level interventions included *Restructuring the social environment*, *Restructuring the physical environment* (when the change was to an existing system), and *Adding objects to the environment* (if something completely new was added to the setting). While coding system level interventions is possible with the BCTTv1, there may be a need to characterise what specifically is ‘restructured’ and/or ‘added’ to be helpful in replication efforts. Future research could explore the underlying themes within these BCTs; describing BCTs within a fully elucidated programme theory could potentially address this issue.

### Limitations and future research

BCT coding depended on reported content, which is a well-known and discussed challenge of using reports as primary data sources [44], particularly in this literature [45]. In particular, our assumption that reporting of clinician education as a strategy would involve at least providing *information about health consequences* of the clinician performing the behaviour and *instruction on how to perform the behaviour* may have resulted in these codes being over-represented. The alternative would have been to miss out on characterising clinician education in some cases, which we deemed to be unhelpful. As more comprehensive checklists such as TIDieR [5] are adopted and taxonomies such as the BCTTv1 are used to describe the active ingredients of implementation interventions in more detail, it will become easier to develop a better, cumulative evidence base.

In typical applications of BCT taxonomies in other literatures, a single behaviour is defined and targeted by the intervention, and the link to BCTs can be assumed to be explicitly related to changing that single behaviour. The reality of the design and reporting of many implementation interventions is that they often target multiple behaviours and outcomes. Thus, we lacked the capacity to explicitly link BCTs to specific behaviours, which may be a limitation. Nevertheless, harnessing the same BCT to change the *multiple* behaviours required to improve care [46] and clinical outcomes may in fact be a strength of applying the BCTTv1 to characterise implementation interventions. Increased clarity in reporting specifically which provider and patient behaviours are targeted for change would help future syntheses to better understand whether the effectiveness of a given strategy and BCT differ according to features of the behaviour itself.

To our knowledge, this was the first attempt to explore the utility and capacity of using the BCTTv1 to code existing implementation interventions for diabetes. We showed that indeed it is possible to use a taxonomy that focuses on behaviour change in this context. There are opportunities for using this approach for coding implementation intervention content in other clinical areas and begin to explore whether BCTs provide the capacity to account for heterogeneity in meta-analyses of implementation intervention effects.

## Conclusion

The BCTTv1 is a useful tool for characterising implementation intervention content in more detail and offers a promising way forward in identifying and analysing the active ingredients of implementation interventions. Prospective use of behaviour change techniques taxonomies for developing and reporting content would further aid in building a cumulative science of effective implementation interventions.

## Additional file

Additional file 1:
**Coding decisions to supplement BCTTv1 definitions and examples.**

## References

[1] Craig P, Dieppe P, Macintyre S, Michie S, Nazareth I, Petticrew M (2008). Developing and evaluating complex interventions: the new Medical Research Council guidance. BMJ..

[2] Davidson K, Goldstein M, Kaplan RM, Kaufmann PG, Knatterud GL, Orleans CT (2003). Evidence-based behavioral medicine: what is it and how do we achieve it?. Ann Behav Med.

[3] Proctor EK, Powell BJ, McMillen JC (2013). Implementation strategies: recommendations for specifying and reporting. Implement Sci..

[4] Michie S, Fixsen D, Grimshaw JM, Eccles MP (2009). Specifying and reporting complex behaviour change interventions: the need for a scientific method. Implement Sci.

[5] Hoffman T, Glasziou PP, Boutron I, Milne R, Perera R, Moher D (2014). Better reporting of interventions: template for intervention description and replication (TIDieR) checklist and guide. BMJ..

[6] McKibbon KA, Lokker C, Wilczynski NL, Ciliska D, Dobbins M, Davis DA (2010). A cross-sectional study of the number and frequency of terms used to refer to knowledge translation in a body of health literature in 2006: a Tower of Babel?. Implement Sci..

[7] Colquhoun H, Leeman J, Michie S, Lokker C, Bragge P, Hempel S (2014). Towards a common terminology: a simplified framework of interventions to promote and integrate evidence into health practices, systems, and policies. Implement Sci..

[8] Michie S, Richardson M, Johnston M, Abraham C, Francis J, Hardeman W (2013). The behavior change technique taxonomy (v1) of 93 hierarchically clustered techniques: Building an international consensus for the reporting of behavior change interventions. Ann Behav Med..

[9] Walshe K (2009). Pseudoinnovation: the development and spread of healthcare quality improvement methodologies. Int J Qual Health Care..

[10] Tricco AC, Ivers NM, Grimshaw JM, Moher D, Turner L, Galipeau J (2012). Effectiveness of quality improvement strategies on the management of diabetes: a systematic review and meta-analysis. Lancet..

[11] Heran BS, Chen JMH, Ebrahim S, Moxham T, Oldridge N, Rees K (2011). Exercise-based cardiac rehabilitation for coronary heart disease. Cochrane Database Syst Rev..

[12] Beckstead JW, Pezzo MV, Beckie TM, Shahraki F, Kentner AC, Grace SL (2014). Physicians’ tacit and stated policies for determining patient benefit and referral to cardiac rehabilitation. Med Decis Mak..

[13] Ghisi GLM, Polyzotis P, Oh P, Pakosh M, Grace SL (2013). Physician factors affecting cardiac rehabilitation referral and patient enrollment: a systematic review. Clin Cardiol..

[14] Lemstra ME, Alsabbagh W, Rajakumar RJ, Rogers MR, Blackburn D (2013). Neighbourhood income and cardiac rehabilitation access as determinants of nonattendance and noncompletion. Can J Cardiol..

[15] Ghisi GL, Abdallah F, Grace SL, Thomas S, Oh P (2014). A systematic review of patient education in cardiac patients: do they increase knowledge and promote health behavior change?. Patient Educ Couns..

[16] Ferrier S, Blanchard CM, Vallis M, Giacomantonio N (2011). Behavioural interventions to increase the physical activity of cardiac patients: a review. Eur J Cardiovasc Prev Rehabil..

[17] Abraham C, Michie S (2008). A taxonomy of behavior change techniques used in interventions. Health Psychol..

[18] Michie S, Ashford S, Sniehotta FF, Dombrowski SU, Bishop A, French DP (2011). A refined taxonomy of behaviour change techniques to help people change their physical activity and healthy eating behaviours: the CALO-RE taxonomy. Psychol Health..

[19] Michie S, Whittington C, Hamoudi Z, Zarnani F, Tober G, West R (2012). Identification of behaviour change techniques to reduce excessive alcohol consumption. Addiction..

[20] Michie S, Hyder N, Walia A, West R (2011). Development of a taxonomy of behaviour change techniques used in individual behavioural support for smoking cessation. Addict Behav..

[21] Dombrowski SU, Sniehotta FF, Avenell A, Johnston M, MacLennan G, Araújo-Soares V (2012). Identifying active ingredients in complex behavioural interventions for obese adults with obesity-related co-morbidities or additional risk factors for co-morbidities: a systematic review. Health Psychol Rev..

[22] Rodrigues A, Sniehotta FF, Araujo-Soares V (2013). Are interventions to promote sun-protective behaviors in recreational and tourist settings effective? A systematic review with meta-analysis and moderator analysis. Ann Behav Med..

[23] Dombrowski S, Sniehotta F, Johnston M, Broom I, Kulkarni U, Brown J, et al. Optimizing acceptability and feasibility of an evidence-based behavioral intervention for obese adults with obesity-related co-morbidities or additional risk factors for co-morbidities: an open-pilot intervention study in secondary care. Patient Educ Couns. in press. 10.1016/j.pec.2011.08.003 21907528

[24] Avery L, Flynn D, van Wersch A, Sniehotta FF, Trenell MI (2012). Changing physical activity behavior in type 2 diabetes: a systematic review and meta-analysis of behavioral interventions. Diabetes Care..

[25] Ivers N, Jamtvedt G, Flottorp S, Young JM, Odgaard-Jensen J, French SD (2012). Audit and feedback: effects on professional practice and healthcare outcomes. Cochrane Database Syst Rev..

[26] Ivers NM, Sales A, Colquhoun H, Michie S, Foy R, Francis JJ (2014). No more ‘business as usual’ with audit and feedback interventions: towards an agenda for a reinvigorated intervention. Implement Sci..

[27] Gardner B, Whittington C, McAteer J, Eccles MP, Michie S (2010). Using theory to synthesise evidence from behaviour change interventions: the example of audit and feedback. Soc Sci Med..

[28] Lorencatto F, West R, Christopherson C, Michie S (2013). Assessing fidelity of delivery of smoking cessation behavioural support in practice. Implement Sci..

[29] Litzelman DK, Slemenda CW, Langefeld CD, Hays LM, Welch MA, Bild DE (1993). Reduction of lower extremity clinical abnormalities in patients with non-insulin-dependent diabetes mellitus: a randomized, controlled trial. Ann Intern Med..

[30] Taylor CB, Houston Miller N, Reilly KR, Greenwald G, Cunning D, Deeter A (2003). Evaluation of a nurse-care management system to improve outcomes in patients with complicated diabetes. Diabetes Care..

[31] Krass I, Armour CL, Mitchell B, Brillant M, Dienaar R, Hughes J (2007). The Pharmacy Diabetes Care Program: assessment of a community pharmacy diabetes service model in Australia. Diabet Med..

[32] Smith SA, Shah ND, Bryant SC, Christianson TJH, Bjornsen SS, Giesler PD (2008). Chronic care model and shared care in diabetes: randomized trial of an electronic decision support system. Mayo Clin Proc..

[33] Goderis G, Borgermans L, Grol R, Van Den Broeke C, Boland B, Verbeke G (2010). Start improving the quality of care for people with type 2 diabetes through a general practice support program: a cluster randomized trial. Diabetes Res Clin Pract Suppl..

[34] Peterson KA, Radosevich DM, O’Connor PJ, Nyman JA, Prineas RJ, Smith SA (2008). Improving diabetes care in practice: findings from the TRANSLATE trial. Diabetes Care..

[35] Gabbay RA, Lendel I, Saleem TM, Shaeffer G, Adelman AM, Mauger DT (2006). Nurse case management improves blood pressure, emotional distress and diabetes complication screening. Diabetes Res Clin Pract..

[36] Hurwitz B, Goodman C, Yudkin J (1993). Prompting the clinical care of non-insulin dependent (type II) diabetic patients in an inner city area: one model of community care. BMJ..

[37] McDermott RA, Schmidt BA, Sinha A, Mills P (2001). Improving diabetes care in the primary healthcare setting: a randomised cluster trial in remote Indigenous communities. Med J Aust..

[38] Ivers NM, Grimshaw JM, Jamtvedt G, Flottorp S, O’Brien MA, French SD (2014). Growing literature, stagnant science? Systematic review, meta-regression and cumulative analysis of audit and feedback interventions in health care. J Gen Intern Med..

[39] Ferlie EB, Shortell SM (2001). Improving the quality of health care in the United Kingdom and the United States: a framework for change. Milbank Q..

[40] Damschroder LJ, Aron DC, Keith RE, Kirsh SR, Alexander JA, Lowery JC (2009). Fostering implementation of health services research findings into practice: a consolidated framework for advancing implementation science. Implement Sci..

[41] French SD, Green SE, O’Connor DA, McKenzie JE, Francis JJ, Michie S (2012). Developing theory-informed behaviour change interventions to implement evidence into practice: a systematic approach using the Theoretical Domains Framework. Implement Sci..

[42] Reed JE, McNicholas C, Woodcock T, Issen L, Bell D. Designing quality improvement initiatives: the action effect method, a structured approach to identifying and articulating programme theory. BMJ Qual Safety. in press. 10.1136/bmjqs-2014-003103 25319412

[43] Taylor MJ, McNicholas C, Nicolay C, Darzi A, Bell D, Reed JE. Systematic review of the application of the plan-do-study-act method to improve quality in healthcare. BMJ Qual Safety. in press. 10.1136/bmjqs-2013-001862 PMC3963536 24025320

[44] Dombrowski SU, Sniehotta FF, Avenell A, Coyne JC (2007). Towards a cumulative science of behaviour change: do current conduct and reporting of behavioural interventions fall short of best practice?. Psychol Health..

[45] Ivers NM, Tricco AC, Taljaard M, Halperin I, Turner L, Moher D (2013). Quality improvement needed in quality improvement randomised trials: systematic review of interventions to improve care in diabetes. BMJ Open..

[46] Presseau J, Hawthorne G, Sniehotta FF, Steen N, Francis JJ, Johnston M (2014). Improving Diabetes care through Examining, Advising, and prescribing (IDEA): protocol for a theory-based cluster randomised controlled trial of a multiple behaviour change intervention aimed at primary healthcare professionals. Implement Sci..

