# Supplementary material for: Using a behaviour change techniques taxonomy to identify active ingredients within trials of implementation interventions for diabetes care
Source: Implement Sci. 2015 Apr 23;10:55. doi: 10.1186/s13012-015-0248-7 (PMC4438476; doi:10.1186/s13012-015-0248-7)
Supplement: Additional file 1: — Coding decisions to supplement BCTTv1 definitions and examples. [file 13012_2015_248_MOESM1_ESM.docx]

**Additional File 1: BCTs in Diabetes Quality Improvement interventions – Coding decisions following coding**

The following is a list of decisions made for coding particular BCTs following discussions of discrepancies between coders. These decisions are designed to be used alongside existing indication from the BCTTv1 appendix to Michie et al 2013 v1. Where BCTs not mentioned, we used the BCTTv1 original coding rules.

**Additional Table 1. Coding rules to supplement BCTTv1 for coding BCTs targeting healthcare professionals**

| **Label** | **Coding rules to supplement BCTTv1** | **Examples** |
| --- | --- | --- |
| 1. **Goals and planning** | | |
| **1.1 Goal setting (behaviour)** | Use content of reported outcomes to decide whether to code *Goal setting (outcome)* and/or *Goal setting (behaviour*). If unclear if one or other, code both (see 1.3)  Consider targets set by clinical practice guidelines (CPGs) as goal setting (behaviour) when it is clear CPGs have been provided to practices with clear behavioural targets. | Goals were based on the ADA recommendations: […] quarterly A1C measurement, biannual lipid measurement, yearly opthalmological and monoﬁlament exam […]ﬂu vaccine (Gabbay 2007) |
| **1.2 Problem Solving** | No additional coding rules beyond BCTTv1. | At each meeting, teams shared data, ideas, and problems and obtained guidance from project staff (O'Connor 2005) |
| **1.3 Goal setting (outcome)** | Use content of reported outcomes to decide whether to code *Goal setting (outcome)* and/or *Goal setting (behaviour)*. If unclear if one or other, code both (see 1.1)  Consider targets set by clinical practice guidelines (CPGs) as goal setting (outcome) when it is clear CPGs have been provided to practices with clear outcome targets**.** | Targets were set at 7% for HbA1c, 130mmHg for SBP and 100mg/dl (Goderis 2010) |
| **1.4 Action Planning** | As described in BCTTv1, only code if at least one of: context, frequency, duration or intensity –– are detailed, otherwise consider *Goal Setting (behaviour) and/or Goal setting (outcome)*. ‘Self-management plan’ and ‘Change plan’ coded as *Goal setting* unless sufficient detail provided to consider as *Action planning.* | No examples encountered |
| **1.5 Review behaviour goal(s)** | Provision of feedback is not sufficient to code. The behaviour goal must be altered, re-set or agreed not to be changed to be coded. | At the next visit, the facilitator and the GPs discussed the extent to which the plans were carried out (Frijling 2002) |
| **1.6 Discrepancy between current behaviour and goal** | No additional coding rules beyond BCTTv1. | The facilitator and the GPs discussed the extent to which the plans were carried out and which aspects of decision making needed further attention (Frijling 2002) |
| **1.7 Review outcome goal(s)** | Provision of feedback is not sufficient to code. The behaviour goal must be altered, re-set or agreed not to be changed to be coded. | No examples encountered |
| **2. Feedback and monitoring** | | |
| **2.1 Monitoring of behaviour by others without feedback** | Must be active and in service of changing behaviour, not simply routine data collection that is already in place. | No examples encountered |
| **2.2 Feedback on behaviour** | Only code when clearly feedback about the HCP’s behaviour. This code implies a monitoring process providing the feedback data. | Audit and review monthly. Provide feedback to improve progress (Peterson, 2008) |
| **2.3 Self-monitoring of behaviour** | Must be active and in service of changing behaviour, and not simply routine data collection that is already in place. Use supporting content to determine whether it is outcome and/or behaviour being monitored. | No examples encountered |
| **2.4 Self-monitoring of outcomes of behaviour** | Must be active and in service of changing behaviour, and not simply routine data collection that is already in place. Use supporting content to determine whether it is outcome and/or behaviour being monitored. | Track process measures, outcomes, and operational activity (Peterson, 2008) |
| **2.5 Monitoring of behaviour by others without feedback** | The individual being monitored must be aware of the monitoring of their behaviour and it must be in service of changing behaviour, and not simply routine data collection that is already in place. Use supporting content to determine whether it is outcome and/or behaviour being monitored. | No examples encountered |
| **2.6 Biofeedback** | Not sufficiently different from *feedback on outcomes of behaviour* in this context; always code as *feedback on outcomes of behaviour* | No examples encountered |
| **2.7 Feedback on outcomes of behaviour** | Only code when clearly feedback about outcomes of the HCP’s behaviour. This code implies a monitoring process providing the feedback data. | the most recent laboratory results were available to both provider and patient at the time of the patient's visit (Hollbrook 2009) |
| **3. Social support** | | |
| **3.2 Social support (practical)** | Someone new (e.g. case manager, pharmacist) or existing (e.g. other team member) facilitates/helps (rather than replaces) an existing HCP to provide their existing care processes. If a new person introduced or existing team member replaces what was already performed, consider coding *restructure social environment*.  If support provided by a HCP that is part of the existing team, only code if it is clearly help that is beyond what they would normally do. | (nurse case manager provided) surveillance of patients, including phone calls to patients, referred patients to a certiﬁed diabetes nurse educator or a dietitian where appropriate (Gabbay 2007) |
| **4. Shaping knowledge** | | |
| **4.1 Instruction on how to perform the behaviour** | Coding in relation to skills acquisition/development  Code algorithms and decision trees (also consider coding as *Adding objects to environment* if material objects or software)  Consider coding when education is provided.  Code when training is provided. | Physicians received an informational flow sheet providing patient-specific risk factors and foot-care practice guidelines for assessment, diagnostic work-up, treatment, and referral recommendations (Litzelman 1993) |
| **4.2 Information about antecedents** | To be an antecedent, there must be a clear and recurring link between the antecedent and the behaviour: the presence of the antecedent must regularly be followed by the behaviour (e.g., a particular social situation, event, cognition) | No examples encountered |
| **5. Natural consequences** | | |
| **5.1 Information about health consequences** | Code when education is provided. In this case, the health consequences are the consequences for the patient due to the HCPs’ behaviour | The second explained the principles of insulin treatment of T2DM patients in general practice (Goderis 2010) |
| **6. Comparison of behaviour** | | |
| **6.1 Demonstration of the behaviour** | No additional coding rules beyond BCTTv1. | shadowed some of the diabetes care managers and physicians treating patients with diabetes (Taylor 2003) |
| **6.2 Social comparison** | No additional coding rules beyond BCTTv1. | All 21 sites were sent their audit results and a comparison with the combined audit result (McDermot 2001) |
| **6.3 Information about others’ approval** | No additional coding rules beyond BCTTv1.  Must be information that is explicitly given (not merely implied)  Includes views and perspectives of colleagues/other healthcare professional staff if and only if explicitly stated | No examples encountered |
| **7. Associations** | | |
| **7.1 Prompt/cues** | Education advice “reinforced” is not a prompt/cue | folders prompted health care providers to ask patients to remove their footwear, to perform foot examinations, and to provide foot-care education at each visit (Litzelman 1993) |
| **7.3 Reduce prompts/cues** | Code when the lengths between follow-up is increasing. Requires at least two instances of prompting to determine length of time and frequency reduction  Also code when the frequency of prompts reduces. The reduction that needs to be clearly stated. | This visit was followed up by regular (initially weekly) phone calls from BAS and a newsletter every eight weeks (McDermot 2001) |
| **8. Repetition and substitution** | | |
| **8.1 Behavioural practice/ rehearsal** | No additional coding rules beyond BCTTv1. | role-playing used to familiarize them with the treatment algorithms (Krein 2004) |
| **9. Comparison of outcomes** | | |
| **9.1 Credible source** | Consider national organisations, national guidelines and respected peers as credible sources. | The resulting messages were brief, fully referenced (including links to longer abstracts that high- lighted methodological quality and results and to the full text of publications), and linked to local (eg, Institute for Clinical Systems Improvement) and national guidelines (Smith 2008) |
| **10. Reward and threat** | | |
| **10.1 Material incentive (behaviour)** | Only code if stated that participants were told that they *would (in the future*) be provided with a valued object if and only if there has been effort/progress towards the behaviour (as stated in the BCTTv1).  If incentive is provided contingent on recruitment of patients into a group that receives anything beyond usual care, code as reward (as this incentivises performance of the intervention protocol and thus the clinician behaviours). Do not code incentive if only for recruitment to usual care. | No examples encountered |
| **10.2 Material reward (behaviour)** | Only code if clearly stated that a reward was provided after there has been effort and/or progress | Physicians received Euro 60 for each included patient (Goderis 2010) |
| **11. Regulation** | | |
| **11.1 Pharmacological support** | Only code if the pharmacological support is specifically designed to help *behaviour change* | No examples encountered |
| **12. Antecedents** | | |
| **12.1 Restructuring physical environment** | Code only if it is a change to an *existing* physical or virtual (ie software/web) structure  If something physically *NEW* is added to the environment that facilitates the behaviour change, code as *Add objects to the environment*. | No examples encountered |
| **12.2 Restructuring the social environment** | This is beyond support; code when the social environment changes to help care being provided. Code when *someone new* takes on responsibility for providing care that was previously provided by someone else, or someone part of the existing team takes on new care responsibilities. Can be someone added to a core team or shifting care to someone outside the core team (e.g. pharmacist).  Also code when remote services are added (e.g. telecare) as this changes the standard social environment for the clinician. | a diabetologist and nurse educator who, on request, saw patients collaboratively with primary care teams in their respective practices (Wagner 2001)  consulted with the patient using real-time video conferencing (Davis 2003) |
| **12.5 Adding objects to the environment** | Code for the addition of new physical or virtual (software) aimed at facilitating behaviour change. If something existing is changed, consider coding to *Restructuring physical environment.* | The systems intervention, designed to direct health care providers' attention to the prevention of patient-specific risk factors, consisted of colorful folders with foot decals to identify intervention patients (Litzelman 1993) |
